# Using interpretable deep learning to model cancer dependencies

**DOI:** 10.1093/bioinformatics/btab137

**Published:** 2021-05-27

**Authors:** Chih-Hsu Lin, Olivier Lichtarge

**Affiliations:** Department of Molecular and Human Genetics; Department of Molecular and Human Genetics; Department of Biochemistry and Molecular Biology; Department of Pharmacology; Computational and Integrative Biomedical Research Center, Baylor College of Medicine, One Baylor Plaza, Houston, TX 77030, USA

## Abstract

**Motivation:**

Cancer dependencies provide potential drug targets. Unfortunately, dependencies differ among cancers and even individuals. To this end, visible neural networks (VNNs) are promising due to robust performance and the interpretability required for the biomedical field.

**Results:**

We design Biological visible neural network (BioVNN) using pathway knowledge to predict cancer dependencies. Despite having fewer parameters, BioVNN marginally outperforms traditional neural networks (NNs) and converges faster. BioVNN also outperforms an NN based on randomized pathways. More importantly, dependency predictions can be explained by correlating with the neuron output states of relevant pathways, which suggest dependency mechanisms. In feature importance analysis, BioVNN recapitulates known reaction partners and proposes new ones. Such robust and interpretable VNNs may facilitate the understanding of cancer dependency and the development of targeted therapies.

**Availability and implementation:**

Code and data are available at https://github.com/LichtargeLab/BioVNN

**Supplementary information:**

Supplementary data are available at *Bioinformatics* online.

## 1 Introduction

Precision medicine aims to improve therapy based on individual patient and disease variations. In cancer, a promising approach is to target treatment on specific genetic vulnerabilities, which encode mechanisms essential to the survival and proliferation of cancer cells. Genetic dependencies differ among cancers and individuals, unfortunately, requiring resource-intensive experimental approaches [e.g. CRISPR screening (Dempster *et al.*, 2019b; Meyers *et al.*, 2017)] to map them. As these experiments are impractical to conduct on every patient, algorithmic methods to pinpoint dependencies may accelerate a general approach to discover cancer essential genes for personalized therapeutic targeting.

One possible approach is deep learning (i.e. neural networks; NNs). It has been useful in biological applications, such as to predict sequence specificities of DNA- and RNA-binding proteins (Alipanahi *et al.*, 2015) and to classify clinical images (Esteva *et al.*, 2017). Despite robust performance, however, these models are like black boxes. Their parameters are difficult to interpret due to their complex and nonlinear relationship with the output (Eraslan *et al.*, 2019). While model interpretability is not uniformly crucial, it is highly desired in biomedical applications, to guide both clinicians and patients to make well-reasoned medical decisions.

To improve interpretability, recent studies sought to encode biological knowledge directly into the architecture of the NN. This led to parameters and output states that represent biological entities or subsystems (Eraslan *et al.*, 2019; Yu *et al.*, 2018). These models were named visible neural networks (VNNs; Ma *et al.*, 2018) as opposed to the traditional, black box NN whose parameters are not interpretable. For example, Ma *et al.* (2018) used Gene Ontology (The Gene Ontology Consortium, 2017) and Clique-eXtracted Ontology (Kramer *et al.*, 2014) to design the architecture of an NN model, DCell, for predicting yeast cell growth given gene deletion genotypes. The same group further extended the model to predict drug responses and synergy (Kuenzi *et al.*, 2020). Lin *et al.* (2017) and Peng *et al.* (2019) embedded protein–protein interactions, protein–DNA interactions and Gene Ontology into VNNs that reduce the dimensions of single-cell RNA-seq data. Eetemadi and Tagkopoulos (2019) used the transcriptional regulatory network to build the architecture of the genetic NN to predict gene expression. These examples suggest that VNNs perform as well or better than traditional NNs and other non-NN methods while providing interpretable models/predictions. However, signaling pathway information has not yet been used to design VNN.

**Fig. 1.**
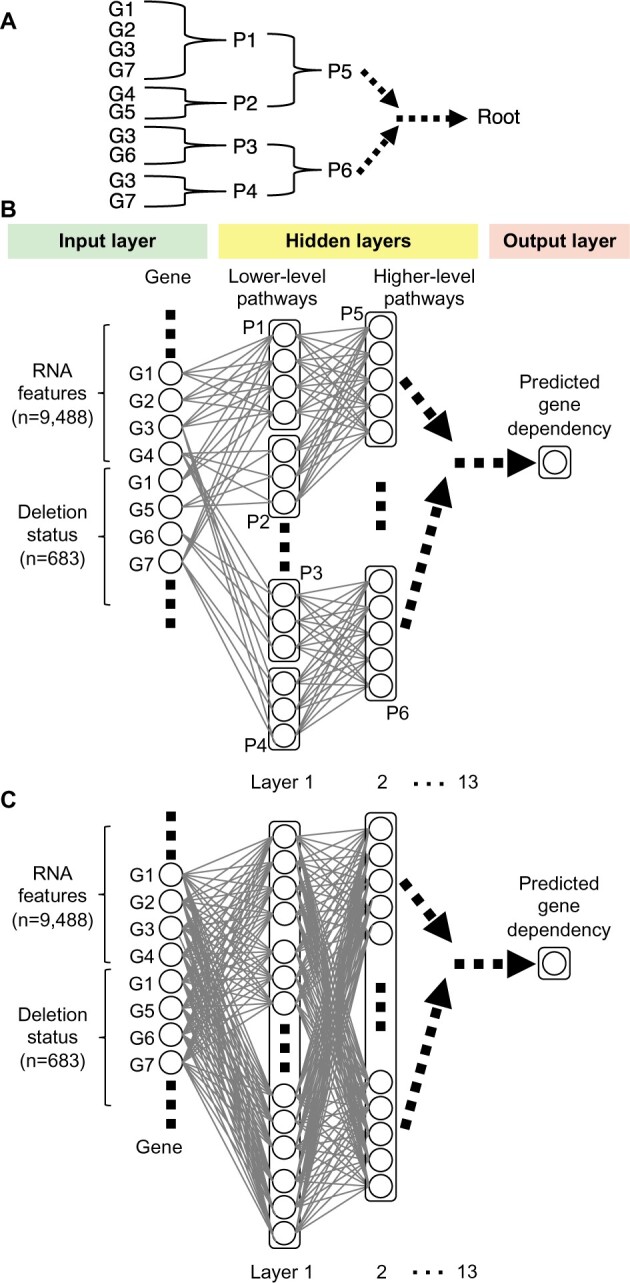
An illustration of the BioVNN for predicting gene dependency. (**A**) A toy example of signaling pathway hierarchy. G, gene; P, pathway. P1 contains G1, G2, G3 and G7; P2 contains G4 and G5; P3 contains G3 and G6; P4 contains G3 and G7; P7 is the parent pathway of P1 and P2; P6 is the parent pathway of P3 and P4. (**B**) A BioVNN designed based on pathway information in (A). The information from nodes in previous layers is integrated in a node of the next layer only if two nodes are connected. The input layer nodes (genes) are connected to the hidden layer nodes (pathways) only when the gene is in that pathway. The input consists of RNA features and deletion status (see Section 2). Lower-level pathways are further connected to higher-level pathways for integrating information toward the prediction of gene dependency. BioVNN is sparsely connected opposed to (**C**) traditional fully connected feedforward network

Pathways [e.g. Reactome (Fabregat *et al.*, 2018)] summarize how some biological components work together to relay upstream signals downstream and biochemically transform molecules through orchestrated series of reactions. Such pathways (Fig. 1A) can be viewed as a hierarchy of interconnected modules that integrate signals and process responses, not unlike an NN architecture (Fig. 1B). Therefore, such pathway information may naturally fit well to build VNNs models that better reflect biological and cellular systems.

**Fig. 2.**
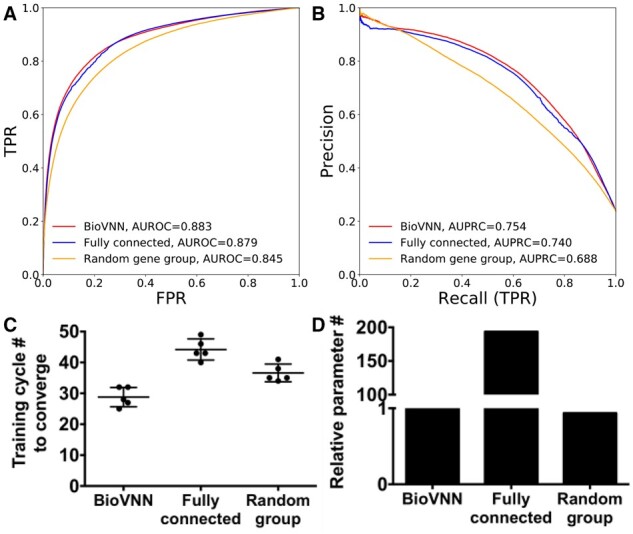
BioVNN predicts the dependency for genes with potential druggability. (**A**) The receiver operating characteristic (ROC) curve and (**B**) precision–recall (PR) curve of BioVNN based on Reactome pathways, the FCN and the matched randomized NN which matches the architecture but with shuffled gene–pathway relationship (random gene groups). The AUROC and the AUPRC were used as the performance metrics (see Section 2). (**C**) The number of training cycles (i.e. epochs) required for the three networks to converge. (**D**) The relative number of trainable parameters of three networks

As a test, VNNs might help deconvolute cancer dependencies where, besides their performance, they could advance the understanding of the internal states of biological systems by linking predictions to underlying mechanisms represented by pathways (Yu *et al.*, 2018). However, to our knowledge, VNNs have not been utilized to predict cancer dependencies to improve the model interpretability and accelerate precision medicine.

To that end, we develop here Biological Visible Neural Network (BioVNN), an interpretable model, to predict the dependencies of potentially druggable genes for cancer cells with RNA expression features. Crucially, the architecture design of BioVNN reflects domain expert-curated signaling pathways, Reactome (Fabregat *et al.*, 2018). Just as convolutional layers combine pixel information of spatial relationships, BioVNN integrates modular information on gene pathways; the neuron units are sparsely connected by following the pathway knowledge (Fig. 1B). We compare BioVNN to a matched random gene group model and to a fully connected network (FCN), and find that even with fewer training cycles, it significantly outperforms the former and slightly outperforms the latter, which has 193 times more parameters in five-fold cross-validation. In a time-stamp experiment, BioVNN outperforms random group model when predicting future observations from prior data. Besides this robust performance, BioVNN predictions can also be explained by the neuron states of specific pathways. The pathway states are different between dependent and nondependent cells and such difference specifically exists only when the gene of target variable is in the pathways. On closer examination, strikingly, BioVNN learns to overweight the feature genes in the same reactions as the genes of target variable, even though such reaction information is never explicitly provided in training data or model. In addition, greater feature weights may suggest novel reaction components. In summary, BioVNN embeds Reactome pathways to predict gene dependency in cancer cells and also provide interpretable neuron states that suggest a rationale for the predictions. By improve our understanding of cancer dependency, this robust and interpretable model is a step toward faster development of precision medicine.

## 2 Materials and methods

### 2.1 Data collection and preprocessing

#### 2.1.1 Pathway data

To design BioVNN from pathway information, the pathways gene set file (*.gmt), pathway hierarchy relationship file, and reaction file were downloaded from Reactome (Fabregat *et al.*, 2018) (https://reactome.org) on May 29, 2019. To ensure that the pathway information was useful, we selected just those 1,425 pathways (including the root) with at least five genes which are feature genes (which expression profiles are used as the model input) and/or genes in the target variable (which dependency predicted in the model output). They consist of total 9,501 genes. The reaction file provides more detailed gene–gene relationship information within a pathway, e.g. binding, activation, translocation, degradation and biochemical events. We test whether the trained models could recapitulate these reaction relationships as validation.

#### 2.1.2 RNA expression features and dependency target variables

We downloaded CCLE RNA expression data of cancer cell lines (Ghandi *et al.*, 2019) and CRISPR data of cancer cell lines (Dempster *et al.*, 2019b; Meyers *et al.*, 2017) of 19Q3 and 20Q2 versions from DepMap (https://depmap.org). In total, 609 cell lines in 19Q3 and 142 different cell lines newly added in 20Q2, which have both expression and CRISPR data, were used.

The expression data are RNA sequencing (RNA-seq) log2-transformed TPM (transcripts per million) values, using a pseudo-count of 1. To select feature genes, we first only considered those with values greater than 1 in at least 1% of the cell lines (i.e. 7 cell lines). Then, we further narrowed our choice to genes present in Reactome. This yielded 9488 genes as RNA expression features.

The CRISPR screening measures the knockout effects of around 18,000 genes on cancer cell growth and the more significant effects represent higher dependencies. The dependency probabilities (between 0 and 1) were used to set up a classification problem of interests, i.e. a gene has a significant effect on a cell line, as suggested on the DepMap webpage: samples with dependency ≥0.5 were defined as the positive class (target variable = 1) and the samples with dependency <0.5 were defined as the negative class (target variable = 0).

To choose genes as target variables (which dependencies were predicted in the model output) we first selected genes that exist in both Reactome and CRISPR data. Then, to focus on genes with sufficient data for training and potential druggabilities, we further restricted our choice to a final set of 683 genes with (i) at least six positive samples; (ii) at least six negative samples; (iii) at least one druggable gene category and at least one chemical interaction in DGIdb (which records drug–gene interactions and potentially druggable genes) (Cotto *et al.*, 2018) (v3.0.2). Note also that we excluded genes which were either nearly all dependent or nearly all nondependent across cell lines as they could lead to overestimates and inaccurate performance evaluation.

### 2.2 BioVNN design

Like the convolutional layers combining pixel information based on spatial relationship and forming higher-level abstraction in deeper layers, BioVNN layers integrate information based on gene–pathway and pathway–pathway relationships and simulate the representations of higher-level pathways in deeper layers. We hypothesize that NNs only need to integrate the information of the genes/pathways which are functionally related to predict dependency. In other words, we specifically look for the correlation among RNA expression and deletion status of all genes in the same pathway and the correlation among pathways having the same parents. Hence, the first hidden layer of lowest-level pathways selectively connects those input genes in the same pathways to the same neuron units, which look for the combinatory effects of expressed and knocked-out genes on cell growth/death. Then, it sends the integrated information to the neurons of corresponding parent pathways until reaching the root. These are important distinctions between BioVNN and FCNs. BioVNN selectively integrates the input based on pathway knowledge, whereas FCNs integrate all information from the previous layer (Fig. 1). Code and data are available at https://github.com/LichtargeLab/BioVNN.

The input of BioVNN consists of two parts (Fig. 1B). The first part is the RNA gene expression profile of the cell line. The second part is the deletion status that specifies which gene is ‘knocked-out’ to simulate its effect on the cell line, inspired by DCell (Ma *et al.*, 2018). With the architecture mimicking the 13-level hierarchy of 1,425 Reactome pathways, BioVNN predicts the dependency of the gene in the cell line specified in the input. Conceptually, BioVNN could be viewed as a sparsely connected feedforward network of 13 hidden layers (Fig. 1B).

Mathematically, we denote the dataset as $D=\left\{\left(x_{i,j},y_{i,j}\right)|x_{i,j}\in R^{k+n},y_{i,j}\in \left\{0,1\right\},i\in \left[1,m\right],j\in \left[1,n\right]\right\}$, where k is the number of RNA expression genes, m is the number of cell lines and n is the number of genes in the deletion status. The data used to compare the output prediction of the model, $y_{i,j}$ is the dependency of the gene j in cell line i. Input $x_{i,j}$ is a concatenated vector of ${\tilde{c}}_{i,j}\in R^{k}$ and $g_{j}\in R^{n}$. To reduce the curse of dimensionality and to focus on biologically relevant genes based on pathway knowledge, while predicting dependency of gene j, we mask the RNA expression vector $c_{i}\in R^{k}$ of cell line i as ${\tilde{c}}_{i,j}$; ${\tilde{c}}_{i,j}=c_{i}\circ u_{j}$, where $u_{j}\in R^{k}$ is a binary vector like a filter to keep genes in the same pathways as gene j (1 = same pathway; 0 otherwise). The genes in $u_{j}$ are selected from the smallest pathways to largest pathways until reaching 100 genes because smaller pathways represent stronger relationship than larger pathways.

We denote the deletion status as $g_{j}$, a one-hot encoding vector of n genes (1 = knocked-out; 0 otherwise). $g_{j}$ assigns the gene for predicting dependency and directs model’s attention to that gene. In this way, we formulate the problem as a single-label binary classification (i.e. agnostic to which genes) instead of a multilabel classification (i.e. treating different genes as separate labels and adding more neurons in the output layers). The model could benefit from more samples (i.e. m×n samples instead of m samples), and the dependency prediction for different genes uses the same set of weights, which assumes the signal integration process through pathway hierarchy is the same for predicting dependency of different genes.

We denote the output neuron state $o_{i,j}^{\left(t\right)}$ of pathway t with input $q_{i,j}^{\left(t\right)}$ as

(1) $$o_{i,j}^{\left(t\right)}=\mathrm{Dropout}\left(\mathrm{BatchNorm}\left(\mathrm{Mish}\left(\mathrm{Linear}\left(q_{i,j}^{\left(t\right)}\right)\right)\right)\right).$$

More concretely, when the lowest pathway t is at the beginning of hierarchy, input $q_{i,j}^{\left(t\right)}$ is the concatenated vector of its gene member input selected from $x_{i,j}$; when pathway t has children pathways, $q_{i,j}^{\left(t\right)}$ is the concatenated vector of the output neuron states of its children pathways.

The Linear transformation in Equation (1) is defined as: $\mathrm{Linear}\left(q_{i,j}^{\left(t\right)}\right)=W_{}^{\left(t\right)}q_{i,j}^{\left(t\right)}+b_{}^{\left(t\right)}$. The weight matrix $W_{}^{\left(t\right)}$ with dimension of $s_{o}^{\left(t\right)}\times s_{q}^{\left(t\right)}$ and bias vector $b_{}^{\left(t\right)}$ with length of $s_{o}^{\left(t\right)}$ are the parameters to learn the representation of pathway t. The length of $o_{i,j}^{\left(t\right)}$ vector, $s_{o}^{\left(t\right)}=\max\left(10,\;\left\lceil 0.3*\mathrm{number}\;\mathrm{of}\;\mathrm{genes}\;\mathrm{in}\;\mathrm{pathway}\;t\right\rceil \right)$ and the length of $q_{i,j}^{\left(t\right)}$ vector is $s_{q}^{\left(t\right)}$. Because the representation of pathways with more gene members may be harder to learn, we set $s_{o}^{\left(t\right)}$ proportionally to the size of pathway t, and 10 is the minimum of $s_{o}^{\left(t\right)}$ for pathways with less than 34 genes.

Mish is the smooth, nonmonotonic and nonlinear activation function, which has been shown to outperform ReLU, Swish and others (Misra, 2019). $\mathrm{Mish}\left(x\right)=x\cdot \tanh\left(\mathrm{softplus}(x)\right)$.

BatchNorm is the normalization of mini-batch during training to reduce the internal covariate shift, which has been shown to achieve higher training rate and reduce overfitting (Ioffe and Szegedy, 2015).

Dropout is a technique to randomly drop neuron units during training, which has shown to reduce overfitting (Srivastava *et al.*, 2014). The Dropout probability is set as 0.5 (Srivastava *et al.*, 2014).

The objective function consists of three parts: (i) the loss of the final output from the root of hierarchy, (ii) the loss of the outputs from other individual pathways and (iii) regularization. The function to be optimized is

(2) $$\frac{1}{mn}\sum_{i=1}^{m}\sum_{j=1}^{n}\mathrm{Loss}\left(\mathrm{Sigmoid}\left(\mathrm{Linear}\left(o_{i,j}^{\left(r\right)}\right)\right),\;y_{i,j}\right)+\;\alpha \sum_{t\neq r}^{}\mathrm{Loss}(\mathrm{Sigmoid}\left(\mathrm{Linear}\left(o_{i,j}^{\left(t\right)}\right)\right),\;y_{i,j})+\lambda {\left\|W\right\|}_{2\;}.$$

Here, r is the root, the highest level of pathway hierarchy after integrating information over other pathway t. Loss is the binary entropy loss function and the negative class was weighted as the ratio of positive sample number to negative sample number in training set. The output prediction is $\mathrm{Sigmoid}\left(\mathrm{Linear}\left(o_{i,j}^{\left(r\right)}\right)\right)\in \left\{0,1\right\}$. The Linear function in Equation (2) transforms the vector $o_{i,j}^{}$ to a scalar. Sigmoid is an exponential function to convert the output scalar to probability.

We include the loss term to compare the output scalar value of each pathway against the $y_{i,j}$, so that every pathway could be auxiliary classifiers to predict dependency on its own and could be optimized as features for parent pathways; α is set as 0.3 to adjust the contribution of the term as previously in GoogLeNet (Szegedy *et al.*, 2014) and DCell (Ma *et al.*, 2018). λ is the L2 regularization factor and set as 1.

### 2.3 Training procedure

We initialize the weights by Kaiming initialization (He *et al.*, 2015). The model is trained with mini-batch (*n* = 2000) by an optimizer combining Rectified Adam (Liu *et al.*, 2019) and Lookahead (Zhang *et al.*, 2019) with learning rate 1E−03, implemented in https://github.com/lessw2020/Ranger-Deep-Learning-Optimizer.

The data [$D=\left\{\left(x_{i,j},y_{i,j}\right)\right\}$, where $x_{i,j}$ is a concatenated vector of RNA expression vector and deletion status; $y_{i,j}$ is the dependency of the gene specified in deletion status in the cell line] is split into five folds with balanced classes and tissue types by cell lines. In each fold of the experiment, data are split into training, validation and test sets by the ratio, 3.2:0.8:1, so that the ratio of training set to validation set and the ratio of training set and validation set to test set are the equal at 4:1. The training set was used for training. The validation set was used to determine the early stopping criterion, which was if the loss did not improve for two epochs. The test set is used to assess model performance. The RNA expression values of all three sets are converted to *z*-scores for each gene by using the mean and standard deviation of the training set.

We focus on testing the NN architecture based on biological signaling pathways to increase model interpretability so the space of hyperparameters is not fully searched. We implemented BioVNN using the PyTorch 1.2 on GTX1080 and RTX2080Ti GPUs.

### 2.4 Time-stamped experiment

To test performance in the most realistic context, we ran time-stamped experiments. We used the models trained on data from DepMap 19Q3. Then we applied these models to predict the dependency for 142 cell lines that were added in 20Q2. To be noted, some cell lines have data in both releases. Even if using 20Q2 data for both training and testing could potentially reduce batch effects affecting the performance, we used the data solely from 19Q3 for training and the data solely from 20Q2 for testing. In this case, it tests the robustness of the model to handle variations from different data versions. One gene, FCGR1A, only exists in 19Q3 but not in 20Q2 dependency data. So only 682 target variable genes were tested in this experiment. The Pearson’s correlation of dependency between 19Q3 and 20Q2 is 0.996 in 609 overlapping cell lines and 682 overlapping genes. The Pearson’s correlation of RNA expression between 19Q3 and 20Q2 is 1.0 in 609 overlapping cell lines and 9488 overlapping genes. It shows slight variations between two data versions.

### 2.5 Performance evaluation

We used area under the receiver operating characteristic curve (AUROC) and area under the precision-recall curve (AUPRC) as metrics. In five-fold cross-validation, the test set predictions from each fold of the five-fold cross-validation were combined to calculate the overall AUROC and AUPRC.

## 3 Results

### 3.1 BioVNN predicts cancer dependencies in potentially druggable genes

Because we aimed to predict drug targets, we trained the model to predict cancer dependencies for potentially druggable genes in five-fold cross-validation (see Methods). As a baseline, we first calculated the performance while making prediction based on the expression values because the expression levels have been shown to be correlated with the dependency (Dempster *et al.*, 2019a). However, our results showed that at least for these druggable genes we selected, the correlation is low (Pearson correlation coefficient = 0.32; Supplementary Fig. 1) and the performance is almost random (AUROC = 0.527; AUPRC = 0.256). For model comparison, we used the matched fully connected network (Fig. 1C) which has the same number of neurons in each hidden layer and the same depth as BioVNN. To examine whether the Reactome-based architecture is useful for predicting gene dependency, we generated the matched random gene group model by shuffling the gene-pathway and pathway-pathway relationship as a control (see Supplementary Methods). In the five-fold cross-validation, Figure 2A and 2B showed that overall BioVNN (AUROC = 0.883; AUPRC = 0.754) marginally outperforms the FCN (AUROC = 0.879; AUPRC = 0.740) but, significantly, converges with one third fewer training cycles (i.e., epochs; Fig. 2C; Mann–Whitney–Wilcoxon (MWW) test two-sided *P* < 5.9E−03) and 193 times fewer trainable parameters (Fig. 2D). In addition, BioVNN also outperforms the random gene group model (AUROC = 0.845; AUPRC = 0.688) with 21% fewer epochs (Fig. 2C; MWW test two-sided *P* < 5.9E−03). Interestingly, in the experiments of gradually increasing randomized network connections, we found that AUPRC decays faster than AUROC (Supplementary Fig. 2). Thus, random sparsity may lead to worse performance. Overall, these data suggest that Reactome pathways provide non-random gene group information which facilitates the training of neural networks and the prediction of cancer dependencies.

Because the FCN has similar performance despite much more parameters, we suspected whether those additional model weights converged to zeroes after training. First, we compared the model weights of first layer to BioVNN and found that FCN has a distribution significantly more enriched at zeroes comparing to BioVNN (Kolmogorov–Smirnov test *P* < 1E−16; Supplementary Fig. 2), which suggests that BioVNN uses much fewer parameters but with higher averaged absolute values of weights (∼0.03) to retain similar amount of information in the data. Next, we asked whether those parameters in FCN connect the feature genes of the same pathway to the same computing neuron, which may rediscover the pathways. Because those neurons in FCN cannot be mapped to pathways directly, we formulated gene groups by applying *k*-means clustering (*k* = 826, the number of the pathways in the first layer of BioVNN) to the PCA-compressed model weights. We found that, surprisingly, 353 out of 826 (42.7%) of gene groups overlapped significantly [hypergeometric test adjusted *P* < 0.1 using BH method (Benjamini and Hochberg, 1995)] with at least one Reactome pathways. We further generated random gene groups matching the sizes of gene group generated based on FCN in 1,600 simulations and found the probability to reach 353 overlapping groups is less than 1E−200 given the simulated exponential distribution (Supplement Fig. 3). The results suggest that the parameters of FCN integrate the gene group information that is similar to Reactome pathways used by BioVNN. In addition, FCN also used other ways of integrating gene information, which may imply new gene groups that are also important for predicting cancer dependency.

**Fig. 3.**
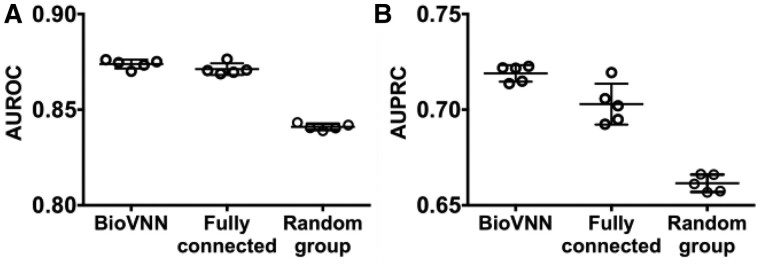
BioVNN predicts the cancer dependency in unseen cell lines of newer data release. Models trained by 19Q3 data were used to predict dependency of cell lines in 20Q2 data. (**A**) AUROC and (**B**) AUPRC showed higher performance in BioVNN and the FCN than the random group model. Each dot is a model trained in one of the five-fold cross-validation

### 3.2 BioVNN predicts dependencies in newer data release

To test predictions prospectively, we setup time-stamped experiments using the training/test dataset from different data releases. We trained the BioVNN on data from DepMap as of 19Q3 (August 2019) and then evaluated its performance over 142 new cell lines that were added later, in the 20Q2 version (May 2020). To be clear, no data from 20Q2 were used for training and 20Q2 data were used for testing. This tests whether the model is robust across data versions given potential batch effects and variations.

Figure 3 shows that, as before, BioVNN marginally gains on the FCN (MWW test two-sided *P > *0.09) and outperforms the random gene group model for both AUROC and AUPRC (MWW test two-sided *P* < 8.0E−03). Again, the results suggest that the pathway knowledge embedded in the BioVNN is helpful to predict cancer dependency. More importantly, by validating the ability of BioVNN to predict dependency prospectively, these data show that BioVNN is generalizable to future data releases.

### 3.3 The neuron states of BioVNN simulate pathway states

Besides performance, interpretability is an essential characteristic of BioVNN. By encoding the Reactome hierarchy, the hidden layers of BioVNN can represent actual signaling pathways whereas traditional NNs or random group models cannot. To determine whether BioVNN’s hidden layers, representing pathways, make predictions more interpretable, we aimed to study why the model made a prediction. More specifically, we investigated three hypotheses. (1) Because the gene dependency is supposed to be affected by its pathway, we hypothesize that when one group of cell lines is dependent and another one is not dependent on the same gene, the output neuron states ($o_{i,j}^{\left(t\right)}$) of the pathways including the same gene from two groups are different. (2) Because other pathways do not include the gene to predict dependency, we hypothesize that we can only find such state difference in only the specific pathways. (3) When one cell line is predicted to be dependent and another one is predicted to be not dependent on the same gene, we hypothesize that the pathway states can suggest key pathways leading to the dependency difference.

**Fig. 4.**
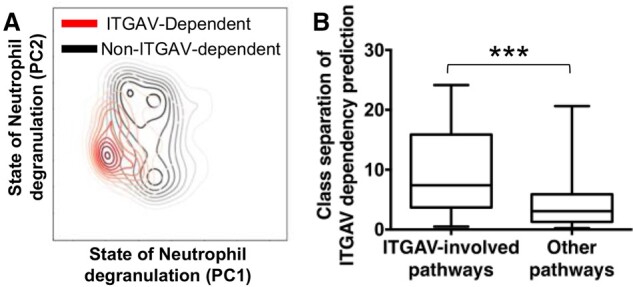
Dependent and nondependent cells have distinct neuron states in related pathways. (**A**) The neuron states of one ITGAV-involved pathway, ‘Neutrophil degranulation’, compressed by principal component analysis to two dimensions and plotted as Gaussian kernel density estimation. PC1 and PC2 are the first and the second principal components. Red and black lines represent significantly different distributions between ITGAV-dependent and non-ITGAV-dependent cell lines (MWW test two-sided *P* < 4.7E−17). (**B**) The class separation of neuron states grouped by whether they consist ITGAV or not. The class separation was measured by how different the PC1 and PC2 of pathway neuron states are between ITGAV-dependent and non-ITGAV-dependent cell lines (see Supplementary Methods). Twenty-seven ITGAV-involved pathways have significantly higher class separation than 1398 other pathways. ***, MWW test one-sided *P* < 6.6E−06

To test the first hypothesis, we took the gene, ITGAV, which is a potential drug target for cancers (Cheuk *et al.*, 2020; van der Horst *et al.*, 2014), as an example. We compared the ITGAV-involved pathway states of ITGAV-dependent cell lines (which have dependency of ITGAV ≥ 0.5) and nondependent cell lines (which have dependency of ITGAV < 0.5). For visualization, we compressed the neuron states of ITGAV-involved pathways by principal component analysis and plotted the first two components with kernel density estimation (Fig. 4A). As an example, the states of the pathway, ‘Neutrophil degranulation’ (R-HSA-6798695), showed significantly different distributions between two classes (i.e. ITGAV-dependent and non-ITGAV-dependent cell lines) (Fig. 4A; combined MWW test two-sided *P* < 4.7E−17; see Supplementary Methods). We found that 26 out of 27 ITGAV-involved pathways show significantly different neuron states between the two classes [combined MWW test two-sided adjusted *P* < 0.1 using Benjamini–Hochberg (BH) method (Benjamini and Hochberg, 1995)]. As a result, the difference of ITGAV dependency could be explained by the state difference of ITGAV-involved pathways.

To test the second hypothesis, we examined whether such class differences are observable in all pathways, i.e. nonspecific, or only in related pathways, which contain the target variable gene (ITGAV). We found that such class separation [as measured by the −log_10_(*P*) value] is significantly larger in related pathways than unrelated pathways (i.e. which do not contain the target variable gene) (Fig. 4C; MWW test one-sided *P* < 6.6E−06). We further expanded the analysis to other genes, and found that in total 74.3% (396 out of 533 target variable genes involved in at least six pathways) have such significant class separation in related pathways [MWW test one-sided adjusted *P* < 0.1 using BH method (Benjamini and Hochberg, 1995)]. That is to say, the dependency differences of the target variable gene between cell lines may be explained by the differences of the neuron states of pathways that contain that target variable gene but not others. Hence, these results suggest that these neuron states could specifically simulate the internal states of pathways in cells and provide explanations for dependency predictions.

**Fig. 5.**
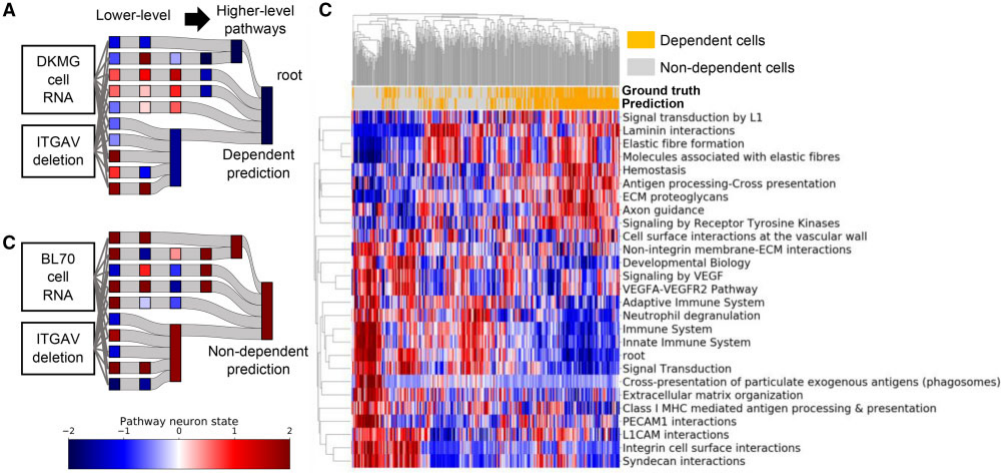
BioVNN explains the dependency by simulated pathway states. (**A**) ITGAV-dependent cell line, DKMG, and (**B**) non-ITGAV-dependent cell line, BL70, showed distinct neuron states of 27 ITGAV-involved pathways in the hierarchy, which explains their reversed predictions. (**C**) The clustered heatmap of neuron states of ITGAV-involved pathways and cell lines. The neuron state colors represent the PC1 of neuron states converted to a *z*-score across cell lines

To test the third hypothesis and further explain the dependency prediction, we examined the neuron states from lower-level to higher-level pathways (Fig. 5A and B). Taking two cell lines, e.g. DKMG (glioma) was predicted to be ITGAV-dependent (Fig. 5A); BL70 (Burkitt lymphoma) was predicted not to be ITGAV-dependent (Fig. 5B). Given their RNA profiles and the ITGAV deletion vectors as input, the neuron states of their ITGAV-involved pathways look distinct (Fig. 5A and B), which explains their opposite predictions. In addition, we further clustered them with other cell lines by the PC1 of their neuron states. We found that predicted ITGAV dependency could be explained by the low neuron states of pathways, which include mostly those under Immune System in the Reactome hierarchy, e.g. Neutrophil degranulation, Innate Immune System and Adaptive Immune System, while non-ITGAV dependency could be explained by high neuron states in these pathways. Hence, these data showed that to predict ITGAV dependency, BioVNN simulated ITGAV deletion and pathway states in the model. The different predictions could be interpreted and explained by the differences in Immune System pathways. It also implies that Immune System pathways may play key roles in ITGAV dependency in cancer cells. These results demonstrate that BioVNN’s predictions are interpretable by inspecting the simulated pathway neuron states.

### 3.4 BioVNN recovers reaction knowledge and suggests new reaction components

Finally, we investigated which features are important for the dependency prediction in BioVNN. We utilized the reaction information from Reactome, which was not used in designing models nor training, to validate whether BioVNN found important features that fit biological knowledge. Reaction information groups genes that are involved in a common process, e.g. binding, activation, translocation, degradation and biochemical events. One gene can be involved in multiple reactions, which are typically smaller gene groups than pathways. A pathway thus often consists of multiple reactions to achieve its function. Two genes involved in the same reaction are in the same pathway but not necessarily vice versa. Such reaction relationships between two genes are stronger than pathway relationships. Should one gene be deleted, its reaction partners are more likely to be affected than pathway partners. Therefore, we hypothesized that even in the same pathway, genes involved in the same reaction as the target variable gene would have higher importance than others.

**Fig. 6.**
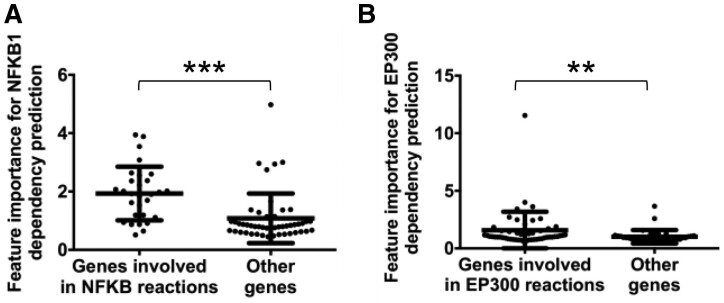
The feature importance of BioVNN recovers the reaction knowledge. (**A**) The feature importance for predicting NFKB1 dependency in the pathway, ‘SASP’ (R-HSA-2559582). (**B**) The feature importance for predicting EP300 dependency in the pathway, ‘Activation of anterior HOX genes in hindbrain development during early embryogenesis’ (R-HSA-5617472). ***, MWW test one-sided *P* < 2.9E−06; **, *P* < 5.0E−04.

Taking NFKB1 as an example, we hypothesized that those genes in the same reactions as NFKB1 have higher feature importance in predicting NFKB1 dependency than other genes in the same pathway. NFKB1 is involved in a total of 53 Reactome reactions, which consist of 333 genes that are NFKB1 reaction partners. One NFKB1-involved pathway at the bottom of the hierarchy is a 78-component pathway, ‘Senescence-Associated Secretory Phenotype (SASP)’ (R-HSA-2559582), consisting of 28 NFKB1 reaction partners. We found that surprisingly, those 28 genes and NFKB1 have significantly higher importance (as measured by feature weights; see Supplementary Methods) than the 49 other genes in the same pathway (MWW test one-sided *P* < 2.9E−06; Fig. 6A). Taking EP300 as another example, EP300 and the 52 genes that are reaction partners with EP300 also have significantly higher feature importance than the other genes in the 88-gene pathway, ‘Activation of anterior HOX genes in hindbrain development during early embryogenesis’ (R-HSA-5617472) (MWW test one-sided *P* < 5.0E−04; Fig. 6B). Furthermore, we expanded the analysis to other target variable genes, and found that 132 out of 1618 gene–pathway pairs (corresponding to 95 of 426 unique genes; see Supplementary Methods) had significantly higher features importance for those genes involved in the same reactions as the target variable gene [MWW test one-sided adjusted *P* < 0.1 using BH method (Benjamini and Hochberg, 1995)]. These results demonstrated that the high-importance features in BioVNN are not random and are in agreement with the biological knowledge of reactions. Of note, only pathway information but not reaction information was used to build the BioVNN architecture. These data suggest that BioVNN recovers the reaction knowledge from the training data on its own even if such knowledge is not provided during model construction or training.

During the analysis above, we found outliers in groups of ‘Other genes’, that were not involved in the same reactions as the target variable gene, that also have high feature importance. In other words, BioVNN regards those genes to be as useful in predicting dependency for the target variable genes as the reaction partner genes. Taking the NFKB1 and SASP pathway as an example (Fig. 6A), the gene of second highest weight in ‘Other genes’ is CDK2, which could suggest a new reaction component with NFKB1. Indeed, a previous study showed that NF-κB bound to the promoter of CDK2, turned on its transcription and upregulated the protein level of CDK2 (Liu *et al.*, 2011). In the example of EP300 (Fig. 6B), the gene of highest weight in ‘Other genes’ is HOXD4, which we propose as a reaction component with EP300. In fact, the protein interaction between HOXD4 and p300 has already been reported (Shen *et al.*, 2001) but is not yet documented in the Reactome database. These findings suggest that genes with high feature importance, that are not reaction partners with the target variable gene, could be candidate reaction components, which have either not been discovered nor added in Reactome database.

## 4 Discussion

Robust and interpretable models are crucial for biomedicine, so we aimed to investigate how pathway knowledge can design VNNs for predicting and interpreting cancer dependency. We have demonstrated the ability of BioVNN to successfully predict cancer gene dependencies and provide interpretable predictions. While converging faster, BioVNN not only significantly outperforms matched random group model but also marginally outperforms the FCN that has 193 times more parameters. BioVNN is also generalizable to predict dependency for cell lines in future releases of the DepMap dataset. By examining the case of ITGAV and overall analysis, we showed that only related pathways have distinct neuron states between dependent and nondependent cell lines whereas most other pathways do not. Specifically, ITGAV dependency could be explained by the low states of pathways related to immune system.

This work illustrates how biological knowledge of signaling pathways can be integrated into an NN architecture. Not only does it solve the issues of designing NN architectures, but also it provides a mechanistic explanation of predictions. For future applications of this work to precision medicine, the RNA-seq expression data of patients could be used to predict personalized cancer dependent genes.

The novel application of signaling pathways to design VNNs was proven to be useful for the first time. In addition, BioVNN uniquely utilizes the pathway-guided feature masks and deletion status vectors to achieve two innovations: (1) training based on bulk RNA-seq data from only hundreds of human cell lines and (2) dependency prediction of hundreds of genes in single model. Given these innovations, a future direction would be to apply VNNs to other cell line data to predict and explain important questions like synthetic lethality and drug responses. The *in silico* states of VNNs could further explain the *in vitro* observations of cell line screenings to synergistically accelerate the development of precision medicine. With more development and validation in the future, these VNNs could be used to predict personalized drug targets and drugs *in vivo* for each patient with interpretable models to explain the predictions and guide their therapies providing better understanding of treatment mechanism.

This study could be expanded in a few ways. First, other biological knowledge could also be embedded in the VNNs, due to the fact that Reactome contains around 10 000 genes, which is only about half of the human protein-coding genes and might limit performance. Many other pathway databases [e.g. Pathway Commons (Rodchenkov *et al.*, 2020), KEGG (Kanehisa and Goto, 2000), MSigDB (Liberzon *et al.*, 2015) and PANTHER (Mi *et al.*, 2019)] could be added to increase the coverage of genes as well as pathway knowledge. In addition, the gene group information can also be nonhuman-curated, such as the gene groups detected from biological networks by computational algorithms (Cantini *et al.*, 2015; Wilson *et al.*, 2017). Since those gene groups are not curated by human, they can be less biased and provide novel functional gene groups.

Second, the model could incorporate more features of cell lines. One possibility is to integrate other types of genomics data besides RNA expression, such as DNA mutation (Ghandi *et al.*, 2019), DNA methylation (Ghandi *et al.*, 2019), copy number variation (Ghandi *et al.*, 2019) and protein expression (Nusinow *et al.*, 2020). These multiple biological observations of the same gene from different angles could be modeled as one state and then be used as features for predicting phenotypes. Another possibility is to incorporate other biological entities [e.g. noncoding RNAs (Ghandi *et al.*, 2019) and metabolites (Li *et al.*, 2019)]. In both ways, the states of the cells could be simulated more precisely and completely.

## Supplementary Material

btab137_Supplementary_Data
