# Supplementary material for: Using interpretable deep learning to model cancer dependencies
Source: Bioinformatics. 2021 May 27;37(17):2675–81. doi: 10.1093/bioinformatics/btab137 (PMC8428607; doi:10.1093/bioinformatics/btab137)
Supplement: btab137_Supplementary_Data [file btab137_supplementary_data.pdf]

Supplementary Data of "Using Interpretable Deep Learning to Model Cancer Dependencies"

Chih-Hsu Lin[1], and Olivier Lichtarge[1,2,3,4,*]

[1] Departments of Molecular and Human Genetics, Baylor College of Medicine, One Baylor Plaza, Houston, TX 77030, USA. [2]Department of Biochemistry and Molecular Biology, [3] Departments of Pharmacology, [4]Computational and Integrative Biomedical Research Center, Baylor College of Medicine, One Baylor Plaza, Houston, TX 77030, USA.

*To whom correspondence should be addressed.

1 Supplementary Methods

1.1 Random gene group model

A random gene group model was generated by shuffling the gene-pathway and pathway-pathway relationships. The size of the random gene group is the same as the corresponding pathway to keep the number of connections as BioVNN. The hierarchy was shuffled by randomly selecting the parent pathway from all pathways that are at the same hierarchy level as the original parent pathway. The same set of hyperparameters and training procedure applied to BioVNN were applied to random gene group model.

1.2 Neuron state analysis

We compress the neuron states of pathways by principle component analysis. We calculate the two-sided Mann–Whitney–Wilcoxon (MWW) test for principle component 1 (PC1) and PC2 between two classes (dependent and non-dependent cell lines) respectively. Then we combine two p-values by Fisher's method (Mosteller and Fisher, 1948) as the final p-value. We further compute the $-\log_{10}(p)$ as the class separation.

1.3 Feature importance analysis

We denote the feature importance of RNA expression of gene k in pathway t as $v_k^{(t)}$:

$$v_k^{(t)} = \sum_{p=1}^{s_q^{(t)}} \left| \boldsymbol{W}_{k,p}^{(t)} \right| \tag{1}$$

where $\boldsymbol{W}_{k,p}^{(t)}$, the p-th scalar value in the k-th vector with length of $s_q^{(t)}$ in the weight matrix $\boldsymbol{W}^{(t)}$, represents the weight of gene k in pathway t. We sum the absolute values of those weights as a measure of feature importance from one model. We calculated the overall importance by summing the feature weights from five models of the 5-fold cross-validation.

When we analyze whether genes in the same reaction as the target variable gene have higher weights than other genes in the same pathway, we only include 1,618 gene-pathway pairs (corresponding to 426 unique genes and 285 unique pathways) which have at least 6 genes in each group to ensure sufficient sample size.

2 Supplementary Figures

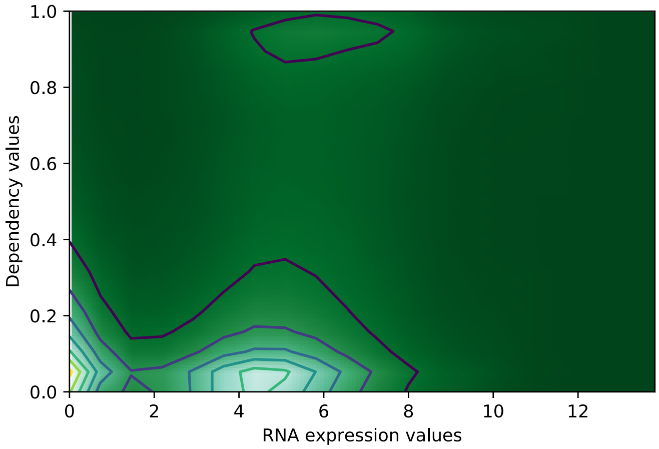

Fig. 1. **Low correlation between RNA expression values and dependency values**. The density plot is based on 621 cell lines and 670 genes that have both RNA expression and dependency data. Lighter color represents higher density. The Pearson correlation coefficient is 0.32.

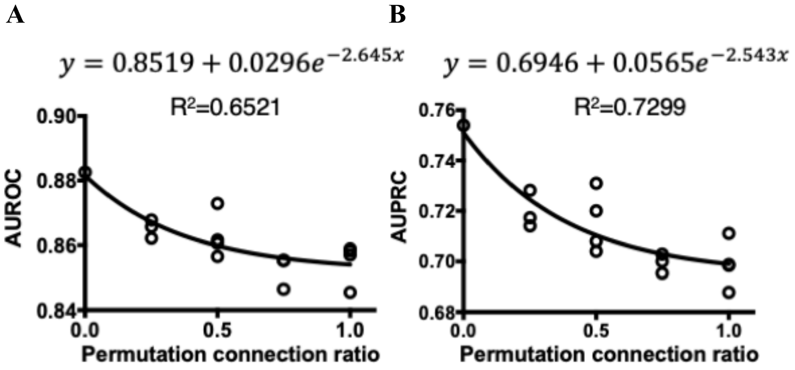

Fig. 2. **Performance decays exponentially while increasing permutated neural network connection ratio**. The BioVNN connections were shuffled at different ratios from 0.25, 0.5, and 0.75 to 1. The partially permutated models were tested in the 5-fold cross-validation. The overall (A) AUROC and (B) AUPRC in the test data were plotted. The curves were fitted as the exponential decay function. The data points at 0 permutation connection ratio were the results of BioVNN.

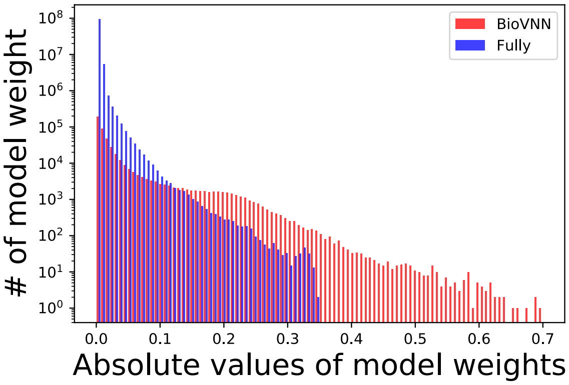

Fig. 3. **BioVNN has higher model weight distribution despite less parameters**. The model weights of first layers were extracted and their absolute values were plotted as the histogram to show their overall distributions.

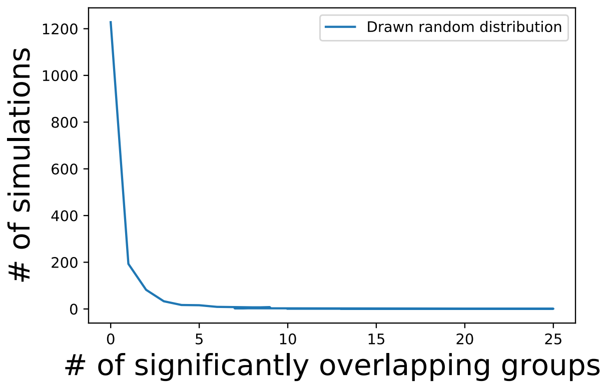

Fig. 4. **Random simulation distribution of numbers of significantly overlapping groups with Reactome pathways**. Each simulation generated a set of random gene groups that matched the size of groups from fully connected network. In the total 1,600 simulations, only one time reached at most 25 significantly overlapping groups with at least one Reactome pathways. Two groups are significantly overlapping when hypergeometric test adjusted $p<0.1$ using Benjamini–Hochberg method (Benjamini and Hochberg, 1995).

References

Benjamini,Y. and Hochberg,Y. (1995) Controlling the False Discovery Rate: A Practical and Powerful Approach to Multiple Testing. *Journal of the Royal Statistical Society. Series B (Methodological)*, **57**, 289–300.

Mosteller,F. and Fisher,R.A. (1948) Questions and Answers. *The American Statistician*, **2**, 30–31.
